# Selective delivery of T22-PE24-H6 to CXCR4^+^ diffuse large B-cell lymphoma cells leads to wide therapeutic index in a disseminated mouse model

**DOI:** 10.7150/thno.43231

**Published:** 2020-04-06

**Authors:** Aïda Falgàs, Victor Pallarès, Naroa Serna, Laura Sánchez-García, Jorge Sierra, Alberto Gallardo, Lorena Alba-Castellón, Patricia Álamo, Ugutz Unzueta, Antonio Villaverde, Esther Vázquez, Ramon Mangues, Isolda Casanova

**Affiliations:** 1 Biomedical Research Institute Sant Pau (IIB-Sant Pau), Hospital de la Santa Creu i Sant Pau, Barcelona Spain.; 2 Josep Carreras Leukaemia Research Institute (IJC), Barcelona, Spain.; 3 CIBER de Bioingeniería Biomateriales y Nanomedicina (CIBER-BBN), Barcelona, Spain.; 4 Institute of Biotechnology and Biomedicine (IBB), Universitat Autònoma de Barcelona, Barcelona, Spain.; 5 Department of Genetics and Microbiology, Universitat Autònoma de Barcelona, Barcelona, Spain.; 6 Department of Hematology, Hospital de la Santa Creu i Sant Pau, Barcelona, Spain.

**Keywords:** targeted nanoparticle, CXCR4 receptor, PE24 exotoxin, DLBCL

## Abstract

**Background**: Novel therapeutic strategies are urgently needed to reduce relapse rates and enhance survival in Diffuse Large B-Cell Lymphoma (DLBCL) patients. CXCR4-overexpressing cancer cells are good targets for therapy because of their association with dissemination and relapse in R-CHOP treated DLBCL patients. Immunotoxins that incorporate bacterial toxins are potentially effective in treating haematological neoplasias, but show a narrow therapeutic index due to the induction of severe side effects. Therefore, when considering the delivery of these toxins as cancer therapeutics, there is a need not only to increase their uptake in the target cancer cells, and their stability in blood, but also to reduce their systemic toxicity. We have developed a therapeutic nanostructured protein T22-PE24-H6 that incorporates exotoxin A from *Pseudomonas aeruginosa,* which selectively targets lymphoma cells because of its specific interaction with a highly overexpressed CXCR4 receptor (CXCR4^+^) in DLBCL.

**Methods**: T22-PE24-H6 cytotoxicity and its dependence on the CXCR4 receptor were evaluated in DLBCL cell lines using cell viability assays. Different* in vitro* experiments (mitochondrial membrane potential, Western Blot, Annexin V and DAPI staining) were conducted to determine T22-PE24-H6 cell death mechanisms. *In vivo* imaging and therapeutic effect studies were performed in a disseminated DLBCL mouse model that mimics organ infiltration in DLBCL patients. Finally, immunohistochemistry and histopathology analyses were used to evaluate the antineoplastic effect and systemic toxicity.

**Results**: *In vitro,* T22-PE24-H6 induced selective cell death of CXCR4^+^ DLBCL cells by activating the apoptotic pathway. In addition, repeated T22-PE24-H6 intravenous administration in a CXCR4^+^ DLBCL-disseminated mouse model showed a significant reduction of lymphoma burden in organs clinically affected by DLBCL cells (lymph nodes and bone marrow). Finally, we did not observe systemic toxicity associated to the nanoparticle treatment in non-DLBCL-infiltrated organs.

**Conclusion**: We have demonstrated here a potent T22-PE24-H6 antineoplastic effect, especially in blocking dissemination in a CXCR4^+^ DLBCL model without associated toxicity. Thereby, T22-PE24-H6 promises to become an effective alternative to treat CXCR4^+^ disseminated refractory or relapsed DLBCL patients.

## Introduction

Over the last years, different immunotoxins that incorporate highly cytotoxic bacterial proteins have entered clinical trials 1,2. Only two of them, moxetumomab pasudotox-tdfk and tagraxofusp-erzs, obtained approval from the FDA for the treatment of hairy cell leukemia (HCL) and blastic plasmacytoid dendritic cell neoplasm (BPDCN), respectively, despite of the induction of severe side effects 3-5. In fact, another immunotoxin (denileukin diftitox), approved to treat patients with cutaneous T-cell lymphoma (CTCL), was withdrawn from the market in 2014 because of life-threatening toxicity 6,7. In this context, clinical translation of antibody drug conjugates (ADCs) and especially immunotoxins is being limited by severe off-target toxicity that leads to a narrow therapeutic window, most likely because only 0.001-0.01% of the injected dose (ID) reaches the tumor 8-10.

Aiming to enhance tumor cell uptake while reducing toxicity on normal tissues, we have developed a multimeric and self-assembled CXCR4- targeted protein-based nanoparticle for the selective delivery of toxins 11. This approach increases toxin loading capacity and the number of targeting ligands per nanoparticle, leading to an increase in nanoparticle internalization and toxin delivery to target cancer cells through its specific receptor 12,13. In our previous work, we have demonstrated that the targeted protein nanocarrier T22-GFP-H6 avoids renal filtration (>7 nm) and liver clearance, whereas most of the nanocarrier proteolytic metabolism occurs in tumor tissues 14,15. In this context, we have generated a stable nanoparticle, T22-PE24-H6, that incorporates the toxin in a single polypeptide. T22-PE24-H6 specifically delivers a de-immunized 24 kDa catalytic domain of *Pseudomonas aeruginosa* (PE) exotoxin A to the chemokine receptor CXCR4 overexpressing (CXCR4^+^) cancer cells by interacting with its T22 ligand 11,16. These cancer cells are relevant clinical targets since CXCR4 overexpression is associated with aggressiveness and dissemination in many solid and hematological cancers 17-21. Indeed, T22-PE24-H6 could improve treatment outcomes in CXCR4^+^ diffuse large B-cell lymphoma (DLBCL) patients, because of their association with poor progression-free as well as overall survival in R-CHOP treated patients; and also because CXCR4^+^ DLBCL cells are responsible for relapse and resistance to R-CHOP 22-24. Currently, no protein-based targeted therapeutic nanoparticle has been developed to treat disseminated or therapy-resistant DLBCL.

**Figure 1:**
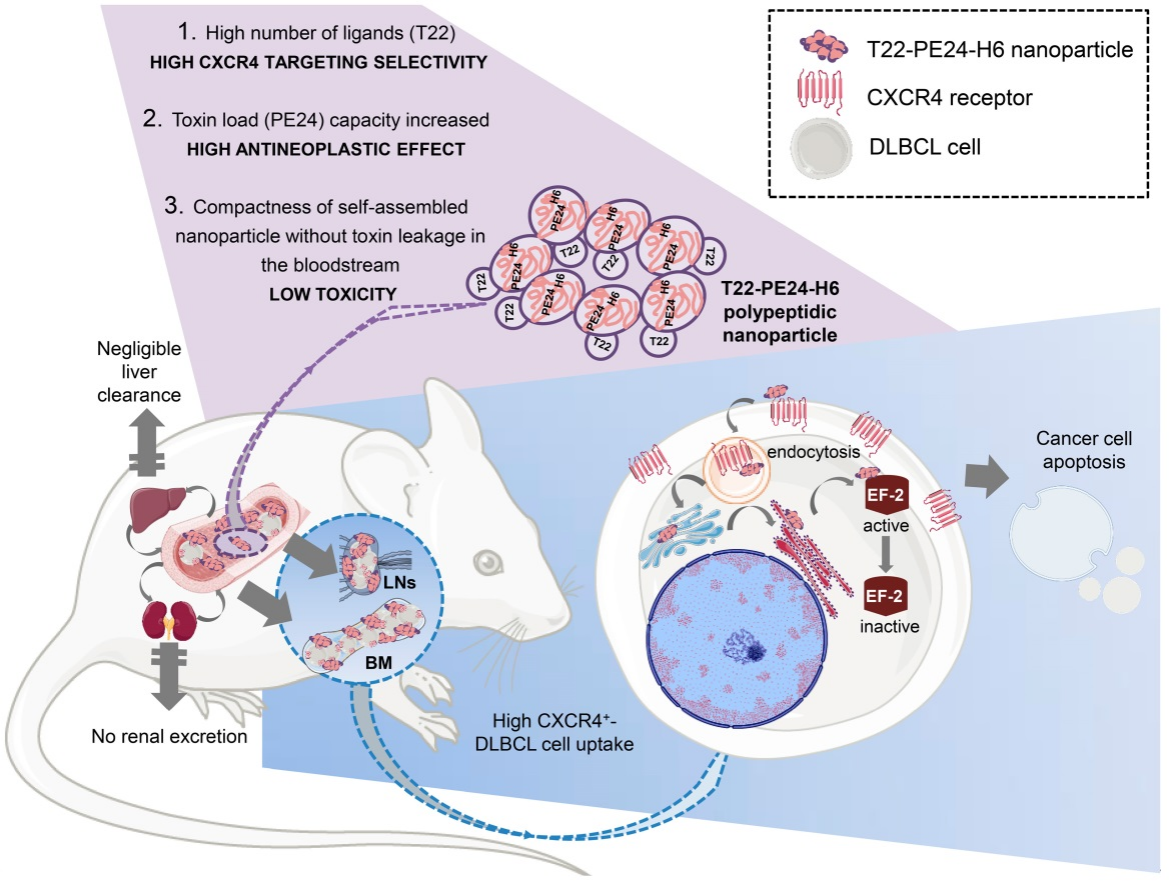
** Graphical image visualizing the highly selective targeting and high cytotoxicity induced by the T22-PE24-H6 nanoparticle on CXCR4^+^ cancer cells in a disseminated DLBCL mouse model.** The image describes critical characteristics of the T22-PE24-H6 polypeptidic nanoparticle that leads to its high CXCR4^+^ DLBCL-cell uptake within LNs and BM. This nanoparticle reaches the neoplastic tissues without being proteolyzed in the liver or excreted by the kidneys. Once in the affected organ, T22-PE24-H6 interacts with the CXCR4 receptor in lymphoma cells, to induce its internalization by endocytosis and its traffic to Golgi and endoplasmic reticulum (ER). There, the PE24 toxin inactivates EF-2, which inhibits protein synthesis and consequently induces cancer cell death by apoptosis. BM: bone marrow; DLBCL: diffuse-large B-cell lymphoma; EF-2: elongation factor 2; LNs: lymph nodes; PE: *Pseudomonas aeruginosa*.

Here, we determine the antineoplastic effect of the T22-PE24-H6 nanoparticle actively targeting CXCR4^+^ DLBCL cells to evaluate whether it could increase the therapeutic window of immunotoxins. Our approach is highlighted in Figure 1. Firstly, we evaluate the *in vitro* cytotoxicity of T22-PE24-H6 in different CXCR4^+^ DLBCL cell lines and its dependence on CXCR4 receptor expression. In addition, we analyze the cell death type induced by T22-PE24-H6 and, most importantly, we evaluate the *in vivo* T22-PE24-H6 antineoplastic effect in DLBCL- infiltrating organs, lymph nodes (LNs) and bone marrow (BM), and its systemic toxicity in a disseminated mouse model. This novel approach aims to increase the cure rates and reduce the toxicity in CXCR4^+^ DLBCL patients.

## Methods

### Production and purification of therapeutic T22-PE24-H6 polypeptidic nanoparticle

All the details of T22-PE24-H6 nanoarchitecture and production were described in our previous work 11.

### Cell culture and cytotoxicity assays

Human DLBCL cell lines (Toledo, SUDHL-6, U-2932 and SUDHL-2) were cultured at 37 ºC in a 5% CO_2_ humidified atmosphere. Toledo, SUDHL-6 and U-2932 cells were cultured in RPMI 1640 medium, and SUDHL-2 cells in IMDM medium. Culture media for all cell lines were supplemented with 10% FBS, 1% glutamine and 100 U/mL penicillin-streptomycin (Life Technologies). Toledo cells were purchased from ATCC, U-2932 and SUDHL-6 from DSMZ, and SUDHL-2 were kindly provided by Dr L. Pasqualucci (Columbia University, NY, USA).

Moreover, the Luciferase plasmid (pPK-CMV- F3, Promokine) was transfected by electroporation (Nucleofector TM 2b Device, Lonza) into Toledo cells (Toledo-Luci cells). The expression of the Luciferase gene in transfected cells was detected by the IVIS Spectrum 200 Imaging System (PerkinElmer). Stable clones were obtained by selection in medium containing 0.2 mg/mL of geneticin (G418, Life Technologies) for a period of 10 weeks.

*In vitro* T22-PE24-H6 cytotoxicity in DLBCL cells was evaluated measuring cell metabolic capacity using the colorimetric cell proliferation kit II (XTT, Roche Diagnostics). 30∙10^4^ cells (Toledo, SUDHL-6, U-2932 and SUDHL-2) were seeded into 96‐well plates in 100 μL of media and incubated at 37 °C for 24 h. Then, cells were exposed to different T22-PE24-H6 concentrations (0.1-5 nM) or carbonate buffer (166 mM NaCO_3_H pH=8) for 48 h and assessed for viability. The competitive assays were done by pre-incubating the cells with AMD3100 (ratio 1T22-PE24-H6:10AMD3100). Fifty μL of the mixture XTT reagent were added to each well and, after 4 h incubation, cell viability was quantified by measuring the absorbance at 450 nm wavelength using a spectrophotometer (BMG Labtech). Results were expressed as percentage of cell viability in relation to its buffer.

### Western blotting

Toledo cells were treated with buffer or 5 nM T22-PE24-H6 for different time exposures (7, 15, 24 and 48 h). Cells were washed twice with PBS and resuspended in lysis buffer. Then, the suspension was sonicated and rested for 20 min on ice. Cells were centrifuged for 10 min at 14000 rpm and 4 ºC. Protein concentration in supernatant was determined using the Bradford protein assay, according to the manufacturer's instructions (BioRad). Cell lysates (50 μg) were separated using 12-15% SDS-PAGE and transferred to a nitrocellulose blotting membrane (GE Healthcare life sciences). Membranes were blocked with 5% skim milk in TBST for 2 h at room temperature, and then incubated with primary antibodies, 1:2000 PARP (556494, BD), 1:1000 caspase-3 (610322, BD) or 1:10000 GAPDH (MAB374, Millipore). Membranes were washed with TBST and then incubated with the appropriate secondary antibody (1:10000, Jackson Immune Research). Western blot visualization was performed using the SuperSignal West Pico Chemiluminescent Substrate (Thermo Fisher Scientific) and the G:BOX iChemi XT Imaging System (Syngene).

Cleaved PARP and active caspase-3 protein levels were quantified using the ImageJ software. The relative density of the protein bands was calculated dividing the percent value of each treated sample (7 h, 15 h, 24 h and 48 h; 5 nM T22-PE24-H6) by the standard sample (Buffer). To get the normalized density value, the relative density of each sample lane was divided by the relative density of its loading control (GAPDH).

### Apoptosis assessment

For the evaluation of JC-1 staining in control and apoptotic cells, the instruction manual of flow cytometry mitochondrial membrane potential detection kit (BD™ MitoScreen Kit) was followed. Results were analyzed by FACS Calibur flow cytometer and the Cell Quest Pro software (BD Biosciences).

Additionally, the number of cells undergoing apoptosis (early or late) was quantified using the Annexin V-FITC/propidium iodide (PI) detection kit (Merck Millipore), following the manufacturer's instructions. Data were analyzed by MACSQuant analyzer flow cytometry using the MACS Quantify version 2.3 software (Miltenyi Biotec).

### DAPI staining

Lymphoma cells were seeded at 3·10^5^ cells/mL (6 mL) in a cell culture dish and incubated at different times (15, 24 and 48 h) with 5 nM T22-PE24-H6 nanoparticle or buffer. Afterwards, cells were centrifuged at 400 g for 5 min, washed with PBS, centrifuged again, resuspended with 1 mL of 3.7% paraformaldehyde and fixed for 10 min at -20 ºC. Then, cells were washed, resuspended with ~10 µL of PBS and placed on a slide. Finally, slides were stained with DAPI mounting medium (Thermo Fisher Scientific) and visualized using a fluorescence microscope (Olympus BX53, Olympus). Representative pictures were taken using an Olympus DP73 digital camera and processed with the cellSens Dimension 1.9 software (Olympus) at 1000 magnifications.

### *In vivo* antineoplastic effect in a DLBCL disseminated mouse model

Four-week-old female NOD-SCID mice were obtained from Charles River Laboratories (L-Abreslle, France), maintained in SPF conditions and housed with sterile food and water *ad libitum. In vivo* experiments were approved by the Hospital de Sant Pau Animal Ethics Committee. After one week in quarantine, mice were intravenously injected with luciferase-transfected Toledo cells (Toledo-Luci cells, 20·10^6^/200 µL) and divided randomly in two groups. Three days after cell injection, one group was administered intravenously with 166 mM NaCO_3_H pH 8 buffer (buffer-treated mice, n=9) and the second group with 5 µg of T22-PE24-H6 (T22-PE24-H6- treated mice, n=9). Both groups were injected three times per week for a total of thirteen doses. Lymphoma dissemination was monitored using bioluminescence imaging (BLI, total radiance photons) technique in the IVIS Spectrum (PerkinElmer), once per week. Mice were anesthetized with 3% isoflurane in oxygen and BLI was captured 5 min after intraperitoneal injection of firefly D-luciferin (2.25 mg/mouse, Perkin Elmer). Moreover, mouse body weight was registered twice a week until the end of the experiment.

All mice were euthanized when the first mouse presented relevant signs of disease such as poor mobility or significant weight loss. At that day, the BLI of lymphoma-infiltrated organs, cervical and renal LNs as well as BM was analyzed *ex vivo*. Finally, all organs were collected and fixed in 4% formaldehyde for further histopathological or immunohistochemical evaluations.

### Cervical LNs size assessment

Cervical LNs area was calculated for 18 cervical LNs in each group (buffer or T22-PE24-H6) using ImageJ software. Area ratio was calculated in relation to buffer-treated LNs.

### Histopathology and immunohistochemical staining

All paraffin-embedded organs were stained with H&E to perform a complete histopathological analysis and a clinical pathologist supervised all samples for toxicity evaluation. To detect the presence of human B-cells and to quantitate the percentage of organ infiltration, we used immunohistochemistry (IHC) staining with an antibody anti-human CD20cy (clone L26, Dako). Firstly, 5 and 27 low power fields at 100 magnifications were taken for LNs and BM, respectively, in each mice group (buffer or T22-PE24- H6). Secondly, the area occupied by B cells in each organ was selected and the percentage of CD20+ cells was quantified using the cellSens Dimension 1.9 software (Olympus).

IHC staining was performed in a DAKO Autostainer Link48 (Agilent) following the manufacturer's instructions. Representative pictures were taken using an Olympus DP73 digital camera and processed with the cellSens Dimension 1.9 software (Olympus) at 200 or 400 magnifications.

### Statistical analysis

All values are expressed as mean ± standard error (SE). Differences between groups were analyzed using the Mann-Whitney U test and were considered statistically significant at p≤0.05. Statistical calculations were performed using SPSS software v21.

## Results

### CXCR4-dependent antineoplastic effect of T22-PE24-H6 in DLBCL cell lines

**Figure 2:**
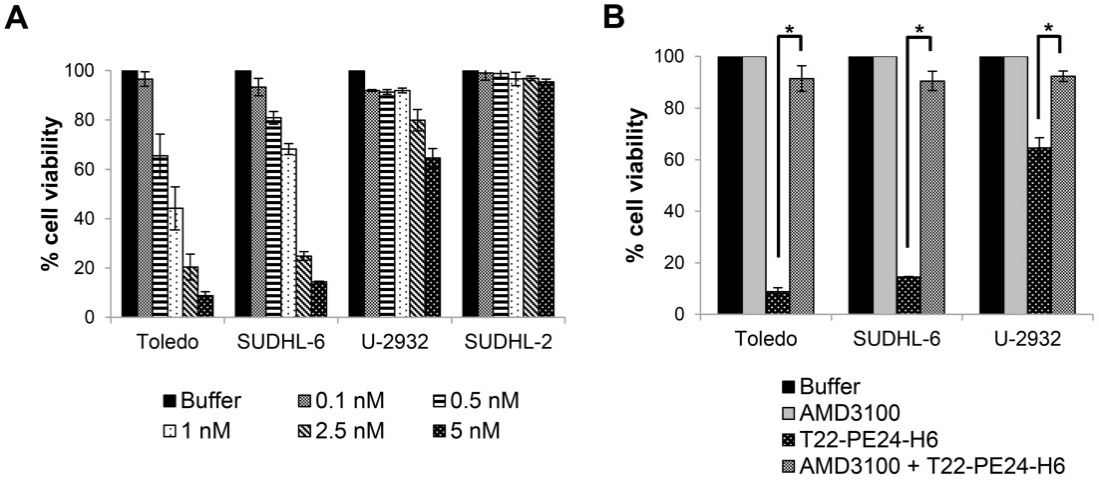
** T22-PE24-H6 induces cytotoxicity in CXCR4^+^ DLBCL cell lines. (A)** *In vitro* cell viability (%) of different DLBCL cell lines after exposure to T22-PE24-H6 (0.1-5 nM) for 48 h. **(B)** Competition cell viability assays done by 1 h pre-treatment with 50 nM AMD3100 followed by the addition of 5 nM T22-PE24-H6 during 48 h exposure in DLBCL cell lines. Toledo, SUDHL-6 and U-2932 are CXCR4^+^ DLBCL cell lines, whereas the SUDHL-2 cell line does not express CXCR4. Experiments were performed in biological triplicates and results expressed as mean ± SE. *p≤0.05.

Levels of CXCR4 membrane expression in the four tested DLBCL cell lines have been previously reported 15,25. Toledo cells showed the highest levels of CXCR4 expression followed by U-2932, SUDHL-6, whereas SUDHL-2 did not express detectable levels of the receptor in their membrane. Thus, we firstly evaluated the sensitivity of these cell lines to the T22-PE24-H6 nanoparticle and its possible correlation with their CXCR4 membrane expression. To that aim, *in vitro* cytotoxic assays were performed after 48 h exposure to different nanoparticle concentrations (0.1-5 nM). Results showed that T22-PE24-H6 therapeutic nanoparticle, at low concentrations, had an antineoplastic effect in Toledo, SUDHL-6 and U-2932 cells, which express high levels of CXCR4 receptor in their membrane (CXCR4^+^ DLBCL cell lines). In contrast, exposure of SUDHL-2 cells (CXCR4^-^ DLBCL cell line) to this nanoparticle did not induce any cytotoxic effect (Figure 2A).

On the other hand, we studied whether the cell death induced by the T22-PE24-H6 nanoparticle in CXCR4^+^ DLBCL cells was dependent on its internalization through the CXCR4 receptor. In this context, competition assays were performed by pre-incubating the cells with AMD3100, a CXCR4 antagonist, 1 h before the addition of T22-PE24-H6, at a molar ratio of 10:1. Cell exposure with AMD3100 blocked nanoparticle binding to CXCR4 and produced a complete reversion of cell viability (~100%) in all three CXCR4^+^ DLBCL cell lines (Figure 2B).

Hence, T22-PE24-H6 displays a potent *in vitro* antineoplastic effect only in the DLBCL cell lines that overexpress CXCR4, since endocytosis of the nanoparticle is exclusively mediated through the CXCR4 receptor.

### T22-PE24-H6 induces apoptosis-mediated cell death in CXCR4^+^ DLBCL cells

**Figure 3:**
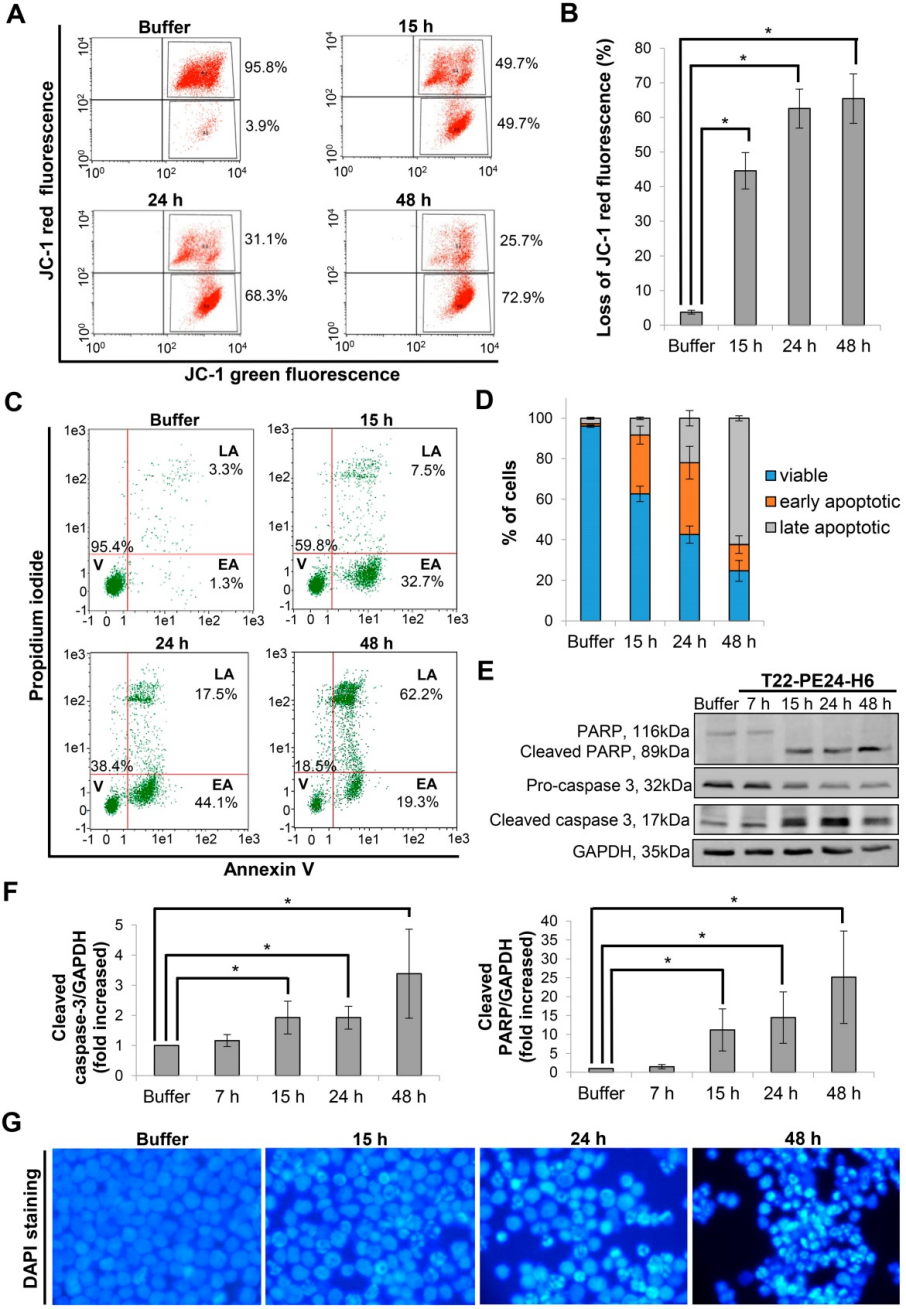
** Apoptosis induction after T22-PE24-H6 exposure in CXCR4^+^ Toledo DLBCL cell line. (A)** Representative dot-plot, showing the percentage of cells undergoing mitochondrial membrane depolarization for this particular replicate (mean percentage is described in the Results text), and **(B)** quantification of loss of JC-1 red fluorescence (%) in Toledo cells after 15 h, 24 h or 48 h exposure to buffer or 5 nM T22-PE24-H6 nanoparticle. **(C)** Representative dot-plot, showing percentages related to this replicate, and **(D)** quantification of viable cells (V), at early or late apoptosis (EA or LA), using Annexin V-FITC/PI test, after 15 h, 24 h and 48 h exposure to 5 nM T22-PE24-H6 or buffer in Toledo cells. **(E)** Toledo cells treated with buffer or 5 nM T22-PE24-H6 for 7 h, 15 h, 24 h and 48 h, and subjected to Western blot using PARP and caspase-3 antibodies. GAPDH antibody is used as an internal control. **(F)** Relative protein intensity quantitation of cleaved caspase-3 and cleaved PARP expression normalized to GAPDH. **(G)** DAPI staining of Toledo cells after exposure to buffer or 5 nM T22-PE24-H6 for 15 h, 24 h and 48 h. Original magnification x1000. Experiments were performed in biological triplicates and results expressed as mean ± SE. *p≤0.05.

After demonstrating the cytotoxic effect of the nanoparticle in CXCR4^+^ DLBCL cells, we performed different assays to identify the mechanism of cell death induction. Firstly, we analyzed whether depolarization of mitochondria was involved as one of the first events in the cell death mediated by T22-PE24-H6 nanoparticle. Thus, we monitored mitochondrial membrane potential by JC-1 dye. We observed that after 15 h exposure of Toledo cells to 5 nM T22-PE24-H6, 44.6±5.3% of cells showed depolarization of mitochondrial membrane and this percentage increased up to 65.5±7.1% at 48 h (Figure 3A-B). Secondly, to evaluate whether the nanoparticle induced cell death through the apoptotic pathway, we performed the AnnexinV-FITC/PI test. We determined that the percentage of cells in early apoptosis increased from 29.0±4.4% at 15 h to 35.5±8.1% at 24 h, decreasing considerably afterwards at 48 h because of a concomitant increase in late apoptosis from 22.0±3.8% at 24 h to 62.4±1.3% at 48 h (Figure 3C-D). Moreover, we used western blot to evaluate the proteolysis of pro-caspase-3 and PARP as markers of apoptosis induction. The exposure of CXCR4^+^ DLBCL cells to T22-PE24-H6, during the 15 h to 48 h exposure period, caused a decrease of the pro-caspase-3 and PARP expression together with the increase of both cleaved caspase-3 and cleaved PARP that occurred at the final stage of cell death (Figure 3E-F). To further confirm apoptosis as the cell death mechanism, we performed nuclear DAPI staining and observed the expected increase of apoptotic bodies over time (Figure 3G).

On this basis, the cell death mechanism induced by T22-PE24-H6 in Toledo CXCR4^+^ DLBCL cell line is mediated by apoptosis activation.

### T22-PE24-H6 antineoplastic effect in the bioluminescent CXCR4^+^ DLBCL disseminated mouse model

**Figure 4:**
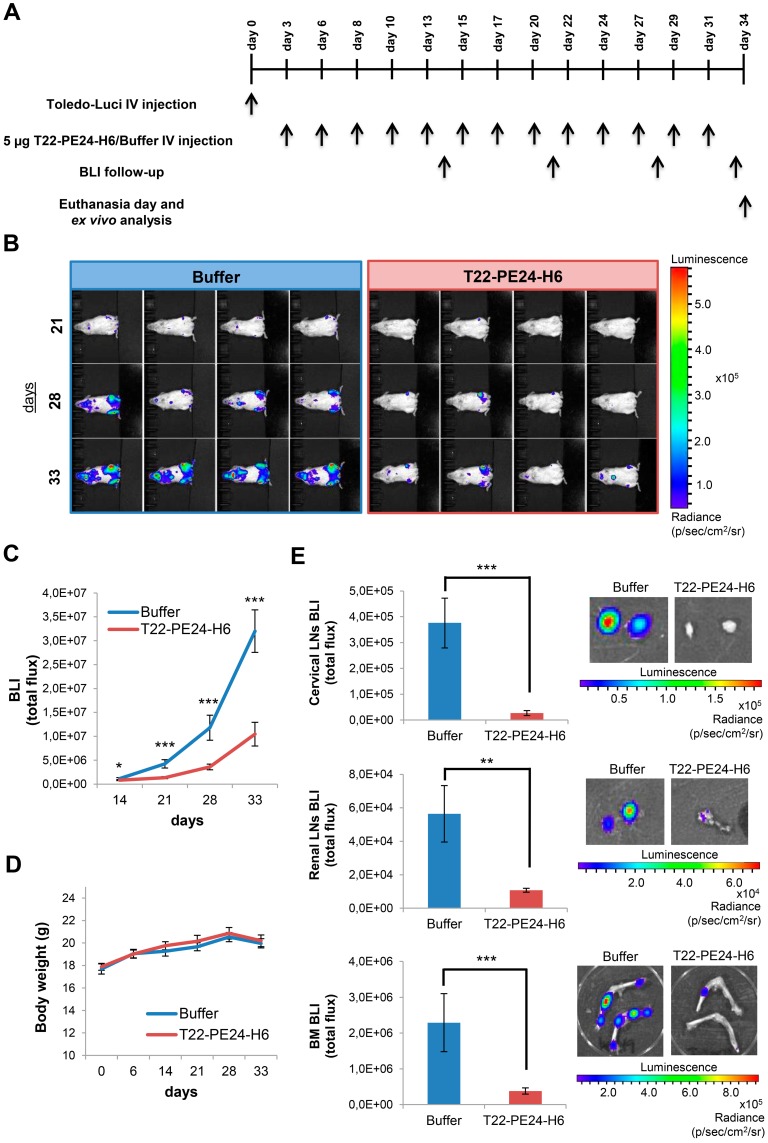
***In vivo* and *ex vivo* BLI analysis of Toledo-Luci disseminated mice treated with buffer or T22-PE24-H6 therapeutic nanoparticle. (A)** Experimental design followed along the *in vivo* experiment. **(B)** Representative images of mice with disseminated lymphoma registered by IVIS Spectrum (BLI signal) at day 21, 28, and 33 after Toledo-Luci cells IV injection in mice treated with buffer or T22-PE24-H6. **(C)** BLI quantification over the experimental time in mice treated with buffer (n=9) or T22-PE24-H6 (n=9). **(D)** Body weight of mice treated with buffer or T22-PE24-H6 during the treatment period. **(E)** *Ex vivo* BLI quantitation (left) and representative images (right) of lymphoma-infiltrated organs (cervical LNs, renal LNs and BM) for both groups at the end of the experiment. Results are expressed as mean ± SE. BLI: bioluminescence imaging (total flux); BM: bone marrow; IV: intravenous; LNs: lymph nodes; *p≤0.05; **p≤0.01; ***p≤0.005.

Afterwards, we also determined the antineoplastic effect of T22-PE24-H6 in a bioluminescent CXCR4^+^ DLBCL disseminated mouse model. This animal model, which was generated by the intravenous injection of Toledo-Luci cells and described in detail in our previous work, shows CXCR4 overexpression in all organs infiltrated by lymphoma cells 15. Here, we started the intravenous administration of 5 µg T22-PE24-H6 or buffer (3 times/week and a total of 13 doses) in this mouse model 3 days after the injection of Toledo-Luci cells. Lymphoma dissemination was monitored until the end of the experiment by capturing the BLI emitted by Toledo-Luci cells using the IVIS Spectrum (Figure 4A). We observed a lower total flux of BLI during the whole follow-up period in nanoparticle-treated mice than in buffer-treated mice. Indeed, this low BLI signal in nanoparticle-treated mice indicated a reduction of lymphoma-cell dissemination, which was already significant at day 14 after cell injection and became highly significant at days 21, 28 and 33 compared to buffer-treated animals (Figure 4B-C). Finally, no significant differences in mouse body weight were found between groups (Figure 4D).

### T22-PE24-H6 nanoparticle antineoplastic effect in CXCR4^+^ DLBCL-infiltrated organs in the disseminated mouse model

At the day of euthanasia (day 34), when the lymphoma was totally disseminated in buffer-treated mice, we analyzed *ex vivo* the bioluminescence emitted by the lymphoma-infiltrated organs in both groups (buffer or T22-PE24-H6). We detected a highly significant antineoplastic effect in the cervical LNs in T22-PE24-H6 treated mice, observing a 14 fold reduction (0.27±0.09E^5^) compared to the BLI emitted in buffer-treated mice (3.76±0.97E^5^). Moreover, the reduction of lymphoma infiltration was observed also in the renal LNs and BM, displaying 5 and 6 times less BLI total flux in the animals treated with the therapeutic nanoparticle (1.07±0.11E^4^ and 0.38±0.09E^6^, respectively) compared to the buffer group (5.64± 1.68E^4^ and 2.29±0.81E^6^, respectively) (Figure 4E).

Thus, a significant reduction in lymphoma dissemination in T22-PE24-H6-treated mice compared to mice treated with buffer is observed in the organs infiltrated by CXCR4^+^ DLBCL cells (LNs and BM).

### T22-PE24-H6 reduces the load of CXCR4^+^ DLBCL lymphoma cells in affected organs

**Figure 5:**
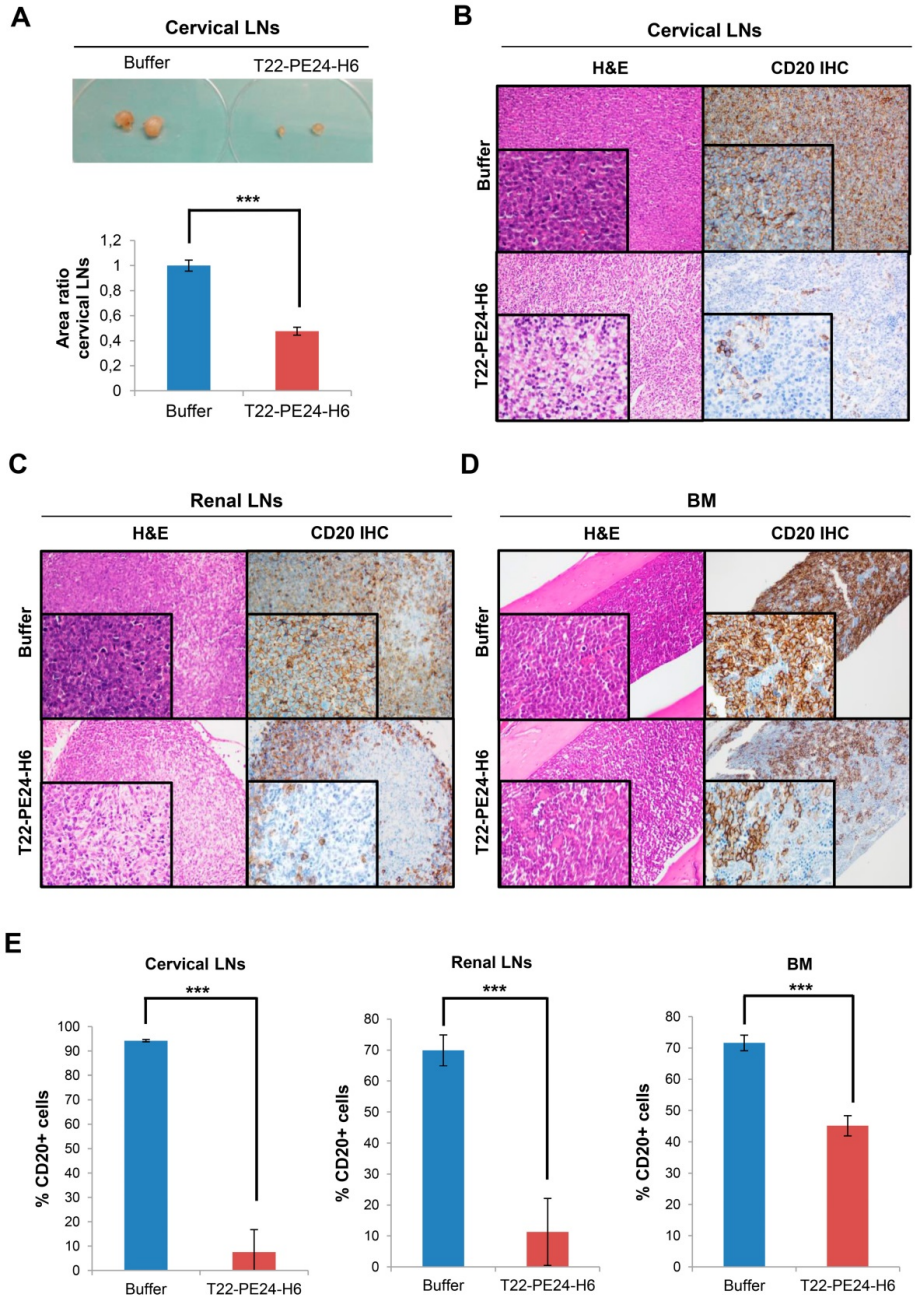
** Macroscopic and microscopic analysis of T22-PE24-H6 antineoplastic effect in the Toledo-Luci disseminated mouse model. (A)** Cervical LNs images in buffer and T22-PE24-H6-treated mice (above) and quantification of cervical LNs area (n=18/group), expressed as area ratio, for both groups (below). **(B-D)** Representative images of H&E staining and CD20 IHC (human DLBCL cells) of lymphoma-infiltrated organs (cervical LNs, renal LNs and BM) in buffer and T22-PE24-H6-treated mice. Original magnification x200 and insets at x400. **(E)** Quantitation of the percentage of CD20+ cells in cervical LNs (n=5 fields/group), renal LNs (n=5 fields/group) and BM (n=27 fields/group) tissues for both mouse groups. Results are expressed as mean ± SE. BM: bone marrow; H&E: hematoxylin and eosin; IHC: immunohistochemistry; LNs: lymph nodes; ***p≤0.005.

Furthermore, we observed macroscopic differences in the cervical LNs size between mice treated with nanoparticle or buffer at the end of the experiment. In this context, the cervical LNs size in nanoparticle-treated mice was significantly reduced more than halfway (47.7±3.2%) compared to the buffer-treated mice (100±4.4%) (Figure 5A). This size reduction was related to the lower number of Toledo-Luci cells infiltrating the cervical LNs, as we demonstrated by IHC using the human B-cell CD20 marker. In buffer-treated mice we found that 94.24± 0.47% of cells within cervical LNs were Toledo-Luci cells (CD20+), whereas the percentage of CD20+ cells diminished significantly in the nanoparticle-treated mice, reaching only 7.53±9.23% (Figure 5B and E). Accordingly, T22-PE24-H6 also induced antineoplastic effect in renal LNs, since the percentage of lymphoma cells was reduced from 69.89±5.01% in mice treated with buffer to 11.31± 10.83% after repeated doses of the nanoparticle (Figure 5C and E). Regarding the BM, we also observed a significant reduction of the Toledo-Luci cells after T22-PE24-H6 treatment. We quantified 71.62±2.50% CD20+ cells infiltrating the BM in buffer-treated mice, a percentage that was reduced to 45.13±3.25% after T22-PE24-H6 treatment (Figure 5D-E).

Definitely, and consistently with BLI detection, we demonstrate the antineoplastic effect of T22-PE24- H6 in CXCR4^+^ DLBCL-infiltrated organs by measuring CD20+ cells in a disseminated mouse model.

### Lack of toxicity by T22-PE24-H6 in the non-infiltrated-DLBCL organs

**Figure 6:**
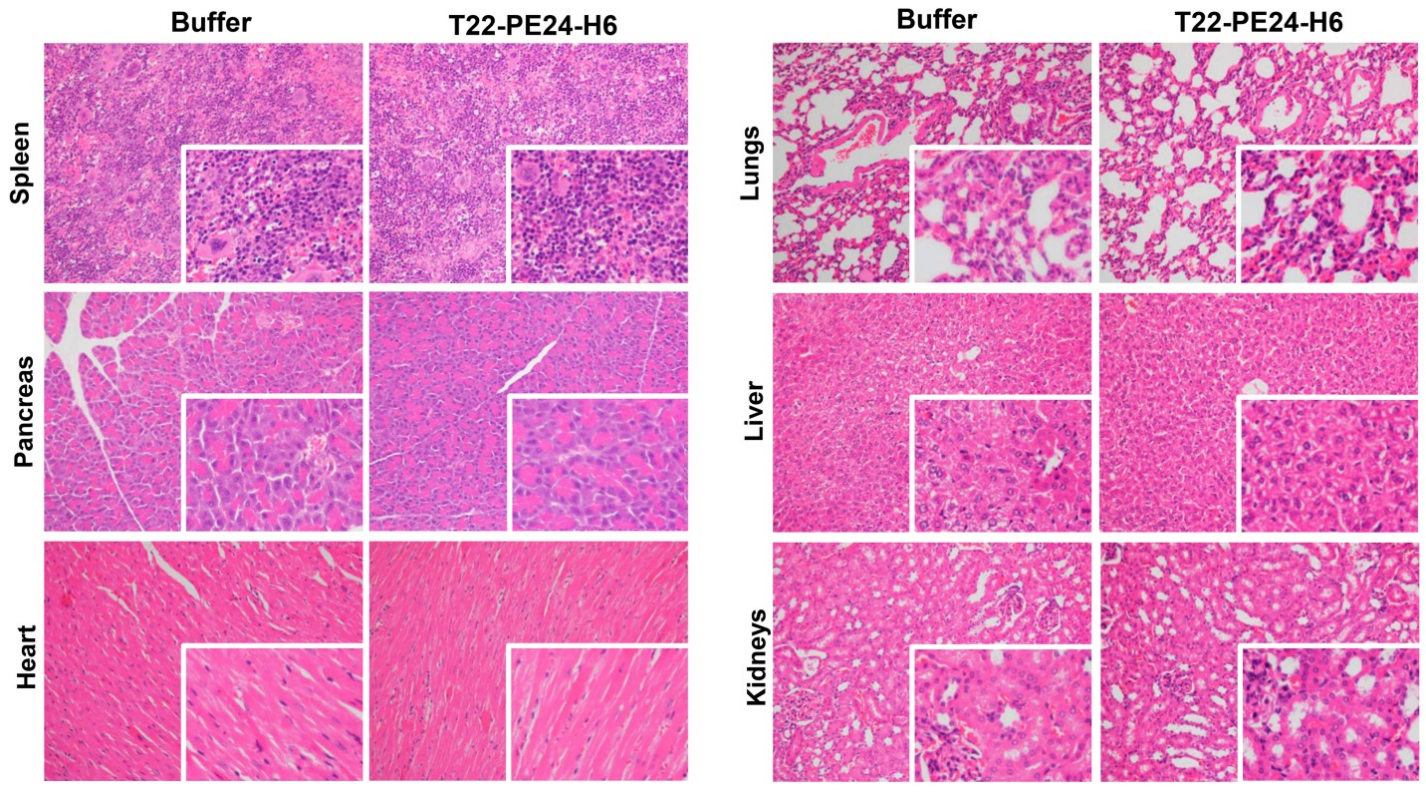
** Toxicity analysis of T22-PE24-H6 treatment in the Toledo-Luci disseminated mouse model.** Histopathology analysis (H&E staining) of non-infiltrated organs (spleen, pancreas, heart, lungs, liver and kidneys) in mice treated with buffer or T22-PE24-H6. Original magnification x200 and insets at x400. H&E: hematoxylin and eosin.

Finally, once we demonstrated the T22-PE24-H6 effectiveness to CXCR4^+^ lymphoma cells we analyzed its harmlessness in non-affected tissues. We did not find any alteration produced by the T22-PE24-H6 nanoparticle at the macroscopic level (data not shown). At microscopic level (Figure 6), we analyzed the histopathology (H&E staining) of non-infiltrated lymphoma tissues in order to carefully assess the possible toxicity of the nanoparticle. Regarding the spleen, we observed the same hematopoietic tissue prevalence in buffer-treated than in nanoparticle- treated mice. Pancreas and heart tissues were also as healthy in nanoparticle-treated as buffer-treated animals. Furthermore, we did not observe congestion, edema or intraalveolar hemorrhage in the lung tissue in nanoparticle-treated mice. In the liver tissue of nanoparticle-treated animals, the hepatocytes did not lose their architecture and we did not observe steatosis or any sign of histological alteration. Finally, the glomerulus and surrounding renal tubules in nanoparticle-treated mice were clearly visible without cytoplasmic vacuolation or eosinophilic protein accumulation. Thus, we did not find any morphological changes associated with nanoparticle administration that could indicate toxicity in non-infiltrated organs.

To sum up, the therapeutic nanoparticle, at the chosen administration conditions, does not induce off-target toxicity in the Toledo-Luci disseminated mouse model.

## Discussion

The administration of the T22-PE24-H6 therapeutic nanoparticle at low doses shows a potent antineoplastic effect in a disseminated DLBCL mouse model without associated toxicity. Both outcomes are achieved by the selective elimination of CXCR4^+^ DLBCL cells, which leads to the control of DLBCL growth and dissemination. This is based on the observation of a lower percentage of CD20+ cells within CXCR4^+^ DLBCL infiltrated organs in nanoparticle-treated animals, while observing no histopathological alteration in non-affected normal organs.

These findings are consistent with our recent demonstration of a high uptake for the T22-GFP-H6 nanocarrier in subcutaneous (SC) tumors generated by CXCR4^+^ lymphoma cells (86.1% of the total ID) and its low or negligible biodistribution to non-DLBCL affected organs (13.9% of the total ID) 15. This high uptake may improve the performance of ADCs, for which less than 1% ID reaches the tumor, while most of the ID is catabolized by the liver and the reticuloendothelial system 26,27. An improved T22-GFP-H6 uptake was also shown in CXCR4^+^ lymphoma cells affecting LNs and BM in a disseminated DLBCL mouse model 15. Indeed, the T22-PE24-H6 nanoparticle size (60.17 nm) and the sinusoidal capillaries present in DLBCL niches (LNs and BM) can profit from the enhanced permeability and retention (EPR) effect 11,28-30. Thus, the high T22-PE24-H6 nanoparticle tumor uptake may take advantage of the EPR effect, the discontinued endothelia in the relevant clinical organs and the active targeting to high CXCR4 overexpressing DLBCL cells.

We argue that the potent antineoplastic effect observed in the absence of toxicity in the disseminated mouse model relates also to the compactness of the self-assembled nanoparticle structure, composed of fusion-protein monomers that incorporate the toxin in a single polypeptide chain, to prevent leakage in the bloodstream 11,31. Regarding this issue, we recently determined that the T22-PE24-H6 protein integrity, and its full-length size (29.2 kDa), is maintained in human blood for at least 10 days 32. Moreover, this exceptional multimeric single polypeptide, which recruits all required functional domains, allows nanoparticle purification in a single step, avoiding the need to chemically conjugate the cytotoxic payload to the carrier while facilitating its fabrication in endotoxin-free bacterial systems 33,34.

Apart from the polypeptidic nanoparticle structure, the constitutive overexpression of the CXCR4 receptor in many available DLBCL cell lines 23,35 and around 30-50% of malignant B-cell lymphocytes derived from DLBCL patients 23,36 allows to achieve a highly selective elimination of aggressive-lymphoma cells by this CXCR4-targeted nanoparticle. Going deeper into the mechanism, T22-PE24-H6 internalizes in CXCR4^+^ tumor cells by endocytosis. Then, the furin-cleavage sites, inserted between the T22 ligand and the functional PE24 toxin, permit toxin intracellular activation in the cytosol, inducing protein synthesis inhibition, which finally leads, as we show here, to the induction of cell death by apoptosis 11,37,38. Interestingly, this mechanism of action permits the killing of dividing and non-dividing cancer cells, differing from the cell cycle-dependent killing induced by genotoxic chemotherapy 2. Consistently, using the same approach, our group previously proved T22-PE24-H6 capacity to selective eliminate CXCR4^+^ colorectal cancer cells in a SC tumor model 11,16.

Regarding the use of PE exotoxin A in current cancer therapies, PE38-based immunotoxins have been tested in clinical trials against different hematological cancers 1, being one of them already approved to treat relapsed or refractory HCL patients 3. However, due to the use of an unstable linkage, the toxin can be prematurely released from the carrier antibody while is circulating in the bloodstream, which together with the immunogenicity induced by the toxin itself limit the dosage that can be used to treat patients 9,39,40. For instance, the US FDA-approved moxetumomab pasudotox-tdfk immunotoxin causes infusion reactions, edema, fever and anemia, among other symptoms, in relapsed or refractory HCL patients. Some of the patients also experience dose-limiting toxicities, mainly hemolytic uremic syndrome and capillary leak syndrome 3. Probably this dosage limitation may underlie the observation that PE38-based immunotoxins targeting different surface markers or receptors expressed in lymphoma cells (e.g. CD22 or CD25) could not induce either partial or complete remissions in non-Hodgkin lymphoma patients 41,42. Currently, many PE38 derivative-based immunotoxins incorporate sequence modifications to reduce their immunogenicity while preserving their efficacy. In this context, PE24 appeared to be less immunogenic than PE38 because of the elimination of several immunogenic B-cell and T-cell epitopes. Accordingly, we incorporated the PE24 toxin to our nanoparticle, as other authors did the same replacement to generate immunotoxins (e.g. PE24-based HA22-LR-8MIT) 7,43.

In conclusion, our novel delivery approach uses a multimeric single polypeptide-based nanoparticle that promises to improve the narrow therapeutic window observed in immunotoxin therapies, especially by reducing the toxicity. Moreover, our results validate CXCR4 overexpressing cells as a relevant clinical target for treating refractory or relapsed DLBCL patients that overexpress CXCR4. Ultimately, future experimental work will dictate the level of relevance of the multiple factors that distinguish protein-nanoparticles from immunotoxins, a requirement to improve immunotoxin or nanoparticle targeting selectivity, which for most current nanoparticles appear to be still limited 44.

## Abbreviations

ADC: antibody-drug conjugate
ATCC: American type culture collection
BLI: bioluminescence imaging
BM: bone marrow
BPDCN: blastic plasmacytoid dendritic cell neoplasm
CTCL: cutaneous T-cell lymphoma
DAPI: 4′,6-diamidino-2-phenylindole
DLBCL: diffuse large B-cell lymphoma
DSMZ: German collection of microorganisms and cell cultures
EPR: enhanced permeability and retention
FACS: fluorescence-activated cell sorting
FBS: fetal bovine serum
FDA: food and drug administration
GAPDH: glyceraldehyde-3-phosphate dehydrogenase
H&E: hematoxylin and eosin
HCL: hairy cell leukemia
ID: injected dose
IHC: immunohistochemistry
IMDM: Iscove's modified Dulbecco's medium
JC-1: 5,6-dichloro-2-[(E)-3-(5,6-dichloro-1,3-diethylbenzimidazol-3-ium-2-yl)prop-2-enylidene]-1,3-diethylbenzimidazole iodide
LNs: lymph nodes
MACS: magnetic-activated cell sorting
PARP: poly(ADP-ribose) polymerase
PBS: phosphate-buffered saline
PE: *Pseudomonas aeruginosa*
R-CHOP: rituximab with cyclophosphamide, hydroxydaunorubicin, oncovin, prednisone
RPMI: Roswell Park Memorial Institute medium
SC: subcutaneous
SDS-PAGE: sodium dodecyl sulfate polyacrylamide gel electrophoresis
SE: standard error
SPF: specific-pathogen-free
TBST: Tris-buffered saline Tween 20
